# Socioeconomic inequalities in the quality of life of older Europeans in different welfare regimes

**DOI:** 10.1093/eurpub/cku017

**Published:** 2014-02-25

**Authors:** Claire L. Niedzwiedz, Srinivasa Vittal Katikireddi, Jill P. Pell, Richard Mitchell

**Affiliations:** 1 Institute of Health and Wellbeing, Public Health, University of Glasgow, Glasgow, UK; 2 Medical Research Council/Chief Scientist Office Social and Public Health Sciences Unit, University of Glasgow, Glasgow, UK

## Abstract

**Background:** Whether socioeconomic inequalities in health and well-being persist into old age and are narrower in more generous welfare states is debated. We investigated the magnitude of socioeconomic inequality in the quality of life of Europeans in early old age and the influence of the welfare regime type on these relationships. **Methods:** Data from individuals aged 50–75 years (n = 16 074) residing in 13 European countries were derived from Waves 2 and 3 of the Survey of Health, Ageing and Retirement in Europe. Slope indices of inequality (SIIs) were calculated for the association between socioeconomic position and CASP-12, a measure of positive quality of life. Multilevel linear regression was used to assess the overall relationship between socioeconomic position and quality of life, using interaction terms to investigate the influence of the type of welfare regime (Southern, Scandinavian, Post-communist or Bismarckian). **Results:** Socioeconomic inequalities in quality of life were narrowest in the Scandinavian and Bismarckian regimes, and were largest by measures of current wealth. Compared with the Scandinavian welfare regime, where narrow inequalities in quality of life by education level were found in both men (SII = 0.02, 95% CI: −1.09 to 1.13) and women (SII = 1.11, 95% CI: 0.05–2.17), the difference in quality of life between the least and most educated was particularly wide in Southern and Post-communist regimes. **Conclusion:** Individuals in more generous welfare regimes experienced higher levels of quality of life, as well as narrower socioeconomic inequalities in quality of life.

## Introduction

Quality of life is an important clinical and societal outcome.^1^^,^^2^ Measuring the quality of life of older people and identifying its determinants is becoming increasingly relevant, as a result of both rising life expectancy and growth in the proportion of the elderly population. There has been a recent shift towards trying to capture subjective quality of life (or well-being), which values how individuals perceive and evaluate their lives. Despite the centrality of well-being to the World Health Organization’s definition of health, defined as ‘a state of complete physical, mental and social well-being and not merely the absence of disease or infirmity’,^3^ research on the social determinants of health has often focussed on negative outcomes such as poor mental health and low self-rated health. The maintenance of quality of life is now recognized by some as a key component of the social contract between governments and the individuals they represent.^4^ However, there is a lack of research on what policies and types of society foster quality of life and are effective at minimizing socioeconomic inequalities in quality of life.

Socioeconomic position has been identified as a key determinant of quality of life, with those currently experiencing socioeconomic disadvantage reporting poorer well-being.^5–7^ Different dimensions of socioeconomic position, such as education and wealth, may have various direct and indirect effects on quality of life. For example, greater material resources in early old age allow individuals to participate in cultural and leisure activities, which may contribute to feelings of self-actualization and autonomy. A higher education level and occupational success could contribute to a more positive evaluation of life and feelings of control over one’s past and future and help provide meaning to life.^8^ It is also likely that effects vary by country due to different welfare state arrangements, which could be considered to moderate the influence of socioeconomic position on quality of life. While the influence of the welfare state on overall levels of quality of life among older people has been explored previously,^9^ there is a lack of systematic research into the role of the welfare state in influencing socioeconomic inequalities in quality of life.

The welfare state refers to the state’s function in providing services, such as education and social insurance, in developed countries.^10^^,^^11^ Public health research often classifies countries into distinct welfare regimes,^12^^,^^13^ based on their institutional arrangements, rules and understandings that direct social policies.^14^^,^^15^ In continental Europe, four distinct welfare regimes have been described: Bismarckian, Scandinavian, Southern and Post-communist. In Bismarckian regimes (including Germany and Switzerland), benefits are often administered by the employer and earnings related, the supportive role of the family is encouraged and social divisions are maintained.^10^ Scandinavian countries are characterized by a highly interventionist state, which aims to promote social equality via the principles of redistribution, universalism, a commitment to full employment and income-protection.^10^^,^^14^ Southern countries (including Spain and Greece) are characterized by fragmented income maintenance schemes and high dependency on the family and voluntary sector.^16^^,^^17^ Post-communist countries (such as Poland and the Czech Republic) are characterized by their transition state and social security systems that contain elements of the Bismarckian regime, but are limited in their overall provision.^18^

No specific part of the welfare system is solely responsible for the maintenance of quality of life,^4^ instead we consider quality of life to be influenced by the collection of all social policies. Our objectives here are to first examine the magnitude of socioeconomic inequalities in the quality of life of Europeans in early old age, using different measures of socioeconomic position. Second, we investigate the influence of the type of welfare regime on the magnitude of socioeconomic inequalities in quality of life.

## Methods

### Data source

Data were taken from Wave 2 (release 2.5.0) and Wave 3 (release 1.0.0) of the Survey of Health, Ageing and Retirement in Europe (SHARE). SHARE is a longitudinal panel survey collected via face-to-face computer-assisted personal interview. Wave 2 was collected during 2006–07 and included representative data from 13 countries. The target population of the second wave comprised all individuals born in 1956 or earlier (and their partners).^19^ The third wave collected retrospective occupational histories during 2008–09. Response and attrition rates for SHARE are detailed elsewhere.^20^ The population in this study included individuals aged 50–75 years who participated in Waves 2 and 3 and were born in their current country of residence (n = 18 324).

### Measurement of quality of life

Quality of life was measured during Wave 2 via CASP-12 (control, autonomy, self-realization and pleasure), a validated measure of positive quality of life in early old age.^21^ The measure contains 12 statements relating to life experiences (online Supplementary box S1), which are summed to produce a generic quality of life scale ranging from 12 to 48, where higher scores reflect higher quality of life. The psychometric properties of CASP have been examined elsewhere.^22^

### Exposure variables

Four measures were used to represent different aspects of socioeconomic position. Education level was recorded using the International Standard Classification of Education (ISCED-97).^23^ Categories were recoded into three levels: low (none, pre-primary, primary and lower secondary education), intermediate (upper and post-secondary education) or high (tertiary education). Individuals missing data for this variable were excluded (N = 274, 1.5%). Occupational information was recorded using major groups of the International Standard Classification of Occupations (ISCO-88 codes) produced by the International Labour Organization and re-coded into a four-level ordinal measure based on skill level.^24^ The respondent’s most recent occupation (aged 50–65 years) was taken from the Wave 3 work history module or previous waves where respondents were asked which occupational group described their most recent job. If the participant had not worked since the age of 50 years, their longest-held job was used if they had worked in the past 15 years. Women who reported never being in paid employment, who had missing values for occupational variables and who worked part-time took the main occupation of their partner, if available. Those still missing occupational information were excluded (N = 1366, 7.5%). Equivalized wealth (in Euros) was derived from the sum of all household financial (e.g. money in bank accounts, stocks or government bonds) and real (e.g. value of own residence or vehicle) assets, minus liabilities (e.g. mortgage or credit card debt). Equivalized income (in Euros) was derived from the annual income of each household. Income and wealth were adjusted for household size using the Organisation for Economic Co-operation and Development method^25^ and adjusted for differences in the purchasing power across countries. Countries were grouped into four welfare regime types: Southern (Greece, Italy and Spain), Scandinavian (Denmark and Sweden), Post-communist (Czech Republic and Poland) and Bismarckian (Austria, Belgium, France, Germany, The Netherlands and Switzerland).

### Statistical analyses

Slope indices of inequality (SIIs) were calculated for the associations between each measure of socioeconomic position and quality of life.^26^ To do this, for each socioeconomic measure, a rank score was assigned to each category (or value for continuous variables) on the basis of the midpoint of their range in the cumulative population distribution (ranked from the lowest to the highest socioeconomic position). The scores were calculated separately by gender, cohort (born pre-1946 or post-1945) and country to take into account the different socioeconomic distributions. The SII is obtained by regressing the outcome on the standardized socioeconomic rank and can be interpreted as the difference in CASP-12 scores between the hypothetically most and least advantaged, taking into account the whole socioeconomic distribution.

Multilevel (random intercept) linear regression models were calculated to investigate associations between socioeconomic position and CASP-12, containing individuals (Level 1) nested within countries (Level 2). Likelihood ratio tests suggested the multilevel models were a better fit compared with single-level regressions. All analyses were stratified by gender, as previous research has demonstrated the relationship between socioeconomic position and quality of life may be different between genders.^27^ All models were controlled for age, in 5-year age bands. In the first step of the modelling strategy, an empty ‘null’ model was run containing the random intercept only. Next, the control variables and the socioeconomic rank of interest were added to the empty model. In the third step, we included welfare regime dummy variables (or fixed effects), using the Scandinavian regime as the reference category. Interaction terms between the socioeconomic position rank and the welfare regime dummy variables were then added. Adjusted mean CASP-12 scores were predicted using Stata’s margins command and graphed to help in the interpretation of results. Age-adjusted single-level regression models stratified by welfare regime were also conducted to help interpret SIIs (online supplementary table S1). Calibrated longitudinal weights to account for unit non-response and sample attrition were used in the descriptive statistics when appropriate.^19^ Missing data for income and wealth were imputed by the SHARE team; further details of the multiple imputation procedure are provided elsewhere.^19^ Individuals with missing outcome data (n = 748) were excluded. Analyses were performed using Stata version 12.1.

## Results

### Overall associations between socioeconomic position and quality of life

The overall sample consisted of 16 074 individuals; 52.5% were female. The highest levels of quality of life were found in the Scandinavian regime and the lowest in the Southern type (table 1). Higher quality of life was mostly found among the advantaged socioeconomic groups compared with the disadvantaged. Looking at the overall association between the different measures of socioeconomic position (table 2), the largest inequalities in quality of life were found by current wealth among both men (SII = 3.91, 95% CI: 3.50–4.31) and women (SII = 3.97, 95% CI: 3.58–4.36). The narrowest inequalities in quality of life were found using occupational position among both genders; the SII was 2.28 (95% CI: 1.82–2.73) for men and 2.67 (95% CI: 2.21–3.12) for women.

**Table 1:** Descriptive statistics for CASP-12 by welfare regime and gender for the measures of socioeconomic position

Variables	Southern				Scandinavian				Post-communist				Bismarckian			
	Men		Women		Men		Women		Men		Women		Men		Women	
	*Mean CASP*	*SD*	*Mean CASP*	*SD*	*Mean CASP*	*SD*	*Mean CASP*	*SD*	*Mean CASP*	*SD*	*Mean CASP*	*SD*	*Mean CASP*	*SD*	*Mean CASP*	*SD*
Education level																
Low	35.4	5.9	33.6	6.2	39.7	4.6	39.8	4.8	34.6	6.3	33.2	6.3	38.6	6.0	37.7	6.1
Medium	36.9	4.8	36.6	5.3	40.9	4.1	39.8	4.6	36.8	5.5	35.6	6.0	38.9	5.5	38.9	5.2
High	37.9	5.5	37.7	5.1	40.2	4.4	40.8	4.2	38.1	5.8	38.2	5.2	39.9	4.9	39.6	4.8
Occupational position																
1 (low)	34.7	6.0	32.7	6.2	39.3	5.1	39.3	5.0	35.6	6.6	33.4	6.2	37.5	6.5	36.6	6.1
2	36.0	5.5	34.8	6.0	40.5	4.3	40.0	4.5	36.0	5.9	34.6	6.3	38.9	5.4	38.7	5.4
3	36.7	5.3	37.0	5.2	39.7	3.8	40.8	4.3	36.0	6.3	35.8	6.4	39.8	5.3	39.8	4.8
4 (high)	38.8	5.1	37.6	5.3	40.7	4.3	40.5	4.5	38.5	4.7	36.9	5.4	40.0	4.9	39.4	5.2
Wealth (quartile^a^)																
1 (low)	34.5	6.0	32.5	6.0	39.1	4.8	38.7	4.9	34.8	6.4	33.0	6.1	37.3	5.7	37.4	6.0
2	35.9	5.5	33.7	6.4	40.6	4.4	40.3	4.7	35.1	6.1	34.2	6.5	38.7	5.7	38.0	5.3
3	36.0	5.6	35.0	6.1	40.5	3.8	40.7	4.3	36.6	5.8	35.5	6.0	39.6	5.0	40.0	4.6
4 (high)	37.7	5.1	36.9	5.2	41.3	3.9	41.3	3.7	38.1	5.0	36.7	5.9	40.8	4.8	39.9	5.2
Income (quartile^a^)																
1 (low)	34.4	5.9	33.1	6.2	39.0	5.1	39.3	4.9	34.3	6.2	33.0	6.1	37.7	5.7	37.6	5.6
2	35.5	5.8	33.5	6.2	40.3	4.2	40.1	4.7	35.9	5.8	34.7	6.4	38.7	5.6	37.9	5.5
3	36.3	5.5	35.4	5.9	40.9	3.8	40.8	4.0	37.0	5.8	35.3	6.3	39.8	5.1	39.8	4.7
4 (high)	37.7	5.0	36.5	5.6	41.1	3.8	40.9	4.1	37.6	5.6	36.7	5.9	40.2	5.2	40.1	5.2
Overall	**36.0**	**5.7**	**34.6**	**6.1**	**40.4**	**4.3**	**40.2**	**4.6**	**36.3**	**6.0**	**34.7**	**6.3**	**39.2**	**5.5**	**38.7**	**5.4**
N	**2219**		**2205**		**1236**		**1386**		**1152**		**1512**		**3028**		**3336**	

N: number of individuals; SD: standard deviation.

a: country-specific.

P < 0.001 for anova test comparing mean CASP scores between welfare regimes for men. P < 0.001 for anova test comparing mean CASP scores between welfare regimes for women.

**Table 2:** Multilevel linear models for CASP-12 containing interaction terms between the welfare regime and measures of socioeconomic position among men (N = 7635) and women (N = 8439)

Variables	Model 1 Education level	Model 2 Occupational position	Model 3 Current wealth	Model 4 Current income
	Regression coefficients (95% CIs)	Regression coefficients (95% CIs)	Regression coefficients (95% CIs)	Regression coefficients (95% CIs)
Men				
Overall SII^a^	2.52 (2.05, 2.98)	2.28 (1.82, 2.73)	3.91 (3.50, 4.31)	3.12 (2.71, 3.52)
Welfare regime^b^				
Southern	−6.77 (−8.86, −4.69)	−5.47 (−7.56, −3.39)	−5.36 (−7.42, −3.31)	−5.17 (−7.23, −3.11)
Post-communist	−6.25 (−8.55, −3.95)	−5.21 (−7.52, −2.91)	−4.93 (−7.20, −2.67)	−5.22 (−7.49, −2.95)
Bismarckian	−1.95 (−3.83, −0.07)	−1.06 (−2.94, 0.81)	−1.49 (−3.34, 0.37)	−1.15 (−3.00, 0.71)
Interactions				
SEP (main effect)	0.02 (−1.09, 1.13)	1.50 (0.41, 2.58)	2.90 (1.90, 3.89)	2.40 (1.40, 3.41)
Southern × SEP	4.23 (2.79, 5.68)	1.64 (0.25, 3.03)	1.40 (0.16, 2.64)	1.01 (−0.24, 2.26)
Post-communist × SEP	3.71 (2.09, 5.33)	1.63 (−0.02, 3.29)	1.07 (−0.37, 2.50)	1.65 (0.21, 3.10)
Bismarckian × SEP	2.04 (0.72, 3.36)	0.26 (−1.03, 1.56)	1.11 (−0.07, 2.29)	0.43 (−0.76, 1.62)
Women				
Overall SII^a^	2.89 (2.43, 3.34)	2.67 (2.21, 3.12)	3.97 (3.58, 4.36)	3.44 (3.04, 3.84)
Welfare regime^b^				
Southern	−8.16 (−10.59, −5.73)	−7.10 (−9.52, −4.68)	−6.75 (−9.15, −4.36)	−6.72 (−9.12, −4.32)
Post-communist	−7.35 (−10.00, −4.70)	−6.29 (−8.94, −3.65)	−5.92 (−8.54, −3.30)	−6.28 (−8.91, −3.66)
Bismarckian	−1.60 (−3.77, 0.57)	−1.03 (−3.20, 1.14)	−1.72 (−3.87, 0.43)	−1.91 (−4.07, 0.24)
Interactions				
SEP (main effect)	1.11 (0.05, 2.17)	2.08 (0.99, 3.16)	3.14 (2.17, 4.10)	2.34 (1.37, 3.32)
Southern × SEP	4.51 (3.06, 5.96)	2.39 (0.99, 3.79)	1.69 (0.46, 2.92)	1.61 (0.37, 2.85)
Post-communist × SEP	3.42 (1.91, 4.93)	1.30 (−0.25, 2.85)	0.54 (−0.79, 1.88)	1.28 (−0.07, 2.62)
Bismarckian × SEP	0.50 (−0.77, 1.77)	−0.64 (−1.94, 0.66)	0.74 (−0.41, 1.89)	1.13 (−0.03, 2.28)

CI = confidence interval; SEP = socioeconomic position.

a: Models contain the SEP rank and age group control variables.

b: Scandinavian regime used as reference category in all models; Models 1 to 4 beneath the overall SII results contain age group control variables (coefficients not shown) and welfare regime dummy variables interacted with the SEP rank.

### Interactions between the welfare regime and socioeconomic position

#### Education level

**Figure 1:**
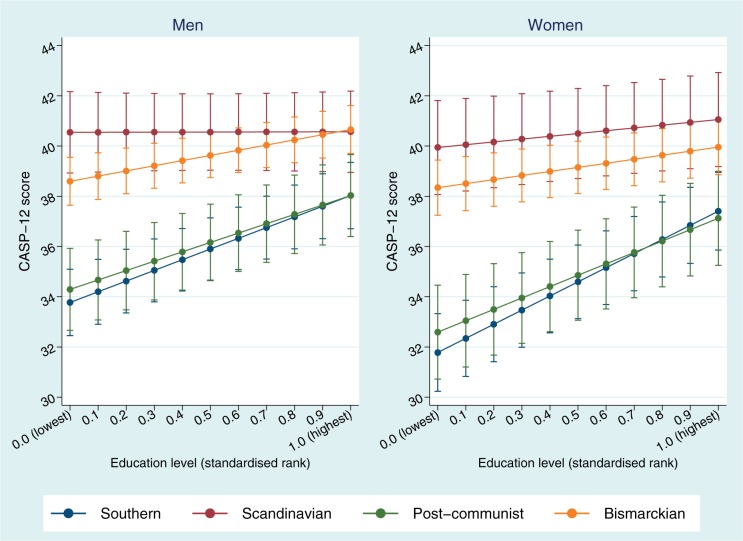
Age-adjusted predicted mean CASP-12 scores for men and women by education level (standardized socioeconomic rank) in different welfare regimes

Among men in the Scandinavian regime, no statistically significant inequalities in quality of life were found for education level (SII = 0.02, 95% CI: −1.09 to 1.13). Compared with the Scandinavian regime, inequalities in quality of life by education level were significantly larger in all other welfare regimes (table 1). The predicted difference in mean quality of life between Scandinavian and Bismarckian regimes diminished as the education rank increased, so that the highest educated experienced very similar levels of quality of life in these two regimes (figure 1). Among women, inequalities in quality of life by education level were small in the Scandinavian regime and Bismarckian regimes, but were particularly large in the Southern and Post-communist regimes.

#### Occupational position

The SII for men in the Scandinavian regime was 1.50 (95% CI: 0.41–2.58) and for women was 2.08 (95% CI: 0.99–3.16). Inequalities in quality of life between the hypothetically highest and lowest occupational positions were largest in the Southern regime among both genders and were of similar magnitude in the Scandinavian and Bismarckian regimes (table 2).

#### Current wealth

**Figure 2:**
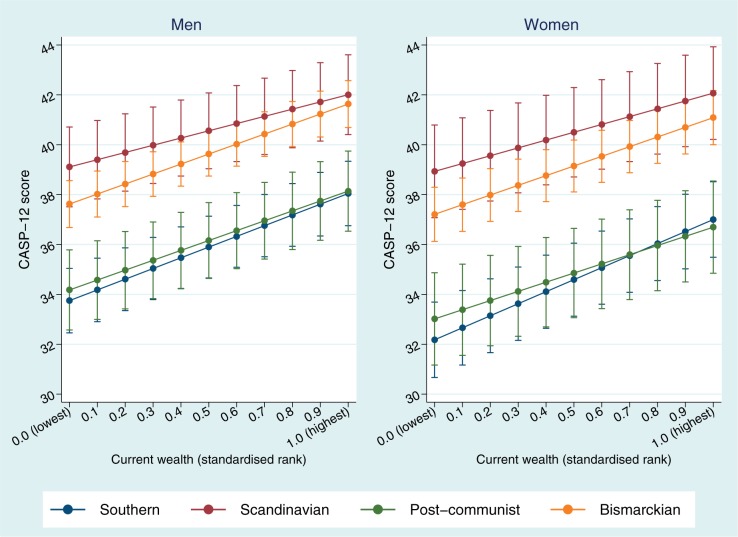
Age-adjusted predicted mean CASP-12 scores for men and women by current wealth (standardized socioeconomic rank) in different welfare regimes

Large inequalities in quality of life by current wealth were found among both genders in all welfare regimes (figure 2). Predicted mean CASP-12 scores for men at the highest end of the wealth distribution in the Southern and Post-communist regimes surpassed those predicted for individuals at the lowest end in the Bismarckian regime. Although the Scandinavian regime exhibited lowest SIIs for wealth among both men (SII = 2.90, 95% CI: 1.90–3.89) and women (SII = 3.14, 95% CI: 2.17–4.10), the gap in quality of life between the most and least wealthy was not much different in the Bismarckian or Post-communist regimes (table 2). Inequalities in quality of life by wealth were largest in the Southern regime among both genders.

#### Current income

The SII for current income among men in the Scandinavian regime was 2.40 (95% CI: 1.40–3.41) and among women was slightly smaller (SII = 2.34, 95% CI: 1.37–3.32). The difference in quality of life between the hypothetically lowest and highest incomes was largest in the Post-communist regime among men and in the Southern regime among women (table 2). There was little difference in the SIIs between the Scandinavian, Bismarckian and Post-communist regimes among women, or between the Scandinavian, Bismarckian and Southern regimes among men.

## Discussion

Socioeconomic inequalities in the quality of life of individuals in early old age were identified in all welfare states, but not using all measures of socioeconomic position. For example, in the Scandinavian regime, no educational inequalities in quality of life were identified among men. We found evidence to suggest the type of welfare regime modified the relationship between socioeconomic position and quality of life. Overall, for most socioeconomic position measures, narrower inequalities in quality of life were found in Scandinavian and Bismarckian countries, and these also displayed the highest levels of quality of life. Educational inequalities in quality of life were small in the Scandinavian regime and largest in Southern and Post-communist countries. Inequalities in quality of life by income and wealth were considerable among all regime types, and there was not much difference in the magnitude of inequalities between regimes. Overall, Southern and Post-communist regimes experienced levels of quality of life which even at the highest end of the socioeconomic scale in most cases did not reach the level of quality of life experienced by those in the lowest position among Scandinavian and Bismarckian regimes.

The observed differences in quality of life can be considered relatively large. Previous studies have quantified effect sizes for CASP by comparing the mean scores for those with and without a limiting illness.^28^ In our sample, the difference in mean CASP scores was 3.5 between those with and without a (self-reported) limiting health problem. This difference was 4.8 for women and 4.0 for men in the Southern regime specifically. In comparison, the difference in quality of life scores between the least and most educated in the Southern regime was around 5.5 for women and 4.1 for men. Thus, the influence of being poorly educated on quality of life in Greece, Italy and Spain was worse than experiencing a limiting illness.

A key strength of our study was the use of high-quality comparable cross-national survey data. Previous studies looking at the relationship between socioeconomic position and health have often relied on occupational cohorts, the results of which may not be generalizable. Research on cross-national variations in health inequalities has also frequently used data from a number of different surveys, and their comparability is questionable.^29^ We also used a theoretically informed outcome measure developed specifically to capture quality of life in early old age, which has been shown to predict 5-year all-cause mortality in older adults.^30^ A number of limitations in our study should be noted. Like all longitudinal panel surveys, SHARE is at risk of attrition and survival bias. However, we suspect this would lead to an underestimation of the observed results. The data were also based on self-reports, which could be affected by differences in language and reporting styles between countries. However, SHARE is subject to rigorous, standardized translation procedures, and previous research has shown that the role of language in explaining cross-national differences in well-being is minimal.^31^ In societies where satisfaction is highly valued, desirability bias may occur if, as a result, individuals are more inclined to report being more satisfied. Some researchers have argued that subjective well-being measures are not contaminated by social desirability, which, if present, may represent a personality trait that influences well-being.^32^

To our knowledge, our study is the first to systematically compare socioeconomic inequalities in the quality of life of older people between welfare states, using a measure of quality of life specifically designed to capture quality of life in early old age. A recent study found similarly narrow socioeconomic inequalities in life satisfaction in Scandinavian and Bismarckian countries, which adds to the strength of our findings.^33^ Research looking at the effects of the welfare state on inequalities in self-rated health (arguably the closest other indicator to quality of life) has had mixed findings. Some studies have found educational inequalities in self-perceived health were smallest in social democratic countries,^34–36^ whereas another study found Scandinavian countries had larger educational inequalities compared with Eastern European countries.^37^ However, self-rated health does not adequately capture positive aspects of well-being, and the determinants of positive health may be different to negative measures of health.^38^

Evidence that socioeconomic inequalities in quality of life are apparent in older ages suggests that inequality ‘gets under the skin’ and into the psychology of people surviving to early old age.^39^ The result that educational inequalities in quality of life are narrowest in the Scandinavian regime may mean that in more egalitarian societies, the experience of living in a more equal society may help individuals to feel in control of their lives and happier about their past and future regardless of their education level. However, the finding that income and wealth inequalities in quality of life are substantial across all welfare states perhaps indicates that in older age, financial resources are most important for well-being, and as welfare states have become less generous in recent years, they may not have been successful at reducing income and wealth inequality and its potentially negative effects.

If growing inequalities in wealth continue,^40^ together with added pressures on the welfare state resulting from demographic change, the financial crisis and austerity policies, they have the potential to widen socioeconomic inequalities across Europe. Our results therefore have important implications for policy. The finding that older people in Scandinavian and Bismarckian welfare regimes have the highest overall well-being and the narrowest socioeconomic inequalities in quality of life suggests that welfare policy could be a key mechanism for addressing inequalities in the well-being of older people.

## Supplementary data

Supplementary data are available at *EURPUB* online.

## Funding

No specific funding was received for this study. At the time of writing, S.V.K was funded by the Chief Scientist Office at the Scottish Health Directorate as part of the Evaluating the Health Effects of Social Interventions programme at the MRC/CSO Social and Public Health Sciences Unit [MC_US_A540_0013]. The funders had no influence over the study design, data collection, analysis, interpretation of data, writing of the paper or decision to submit. The paper does not necessarily represent the views of the funding or employing organization.

*Conflicts of interest*: None declared.

**Key points**

- There is growing policy and research interest in the maintenance and improvement of quality of life, an important aim of welfare states.
- Whether socioeconomic inequalities in health and well-being persist into old age and are narrower in more generous welfare states is debated.
- We found that socioeconomic inequalities in the quality of life of older people were smallest in Scandinavian and Bismarckian regimes using a number of measures of socioeconomic position.
- This suggests that welfare policy may have important implications for both overall quality of life and inequalities in quality of life among older people.

## Supplementary Material

Supplementary Data
