# Supplementary material for: Socioeconomic inequalities in the quality of life of older Europeans in different welfare regimes
Source: Eur J Public Health. 2014 Feb 25;24(3):364–70. doi: 10.1093/eurpub/cku017 (PMC4032483; doi:10.1093/eurpub/cku017)
Supplement: Supplementary Data [file supp_24_3_364__index.html]

Socioeconomic inequalities in the quality of life of older Europeans in different welfare regimes — Socioeconomic inequalities in the quality of life of older Europeans in different welfare regimes — Supplementary Data 

# Socioeconomic inequalities in the quality of life of older Europeans in different welfare regimes

## Supplementary Data

files

**Files in this Data Supplement:**

- Supplementary Data - doc file
