# Supplementary material for: Socioeconomic inequalities in the quality of life of older Europeans in different welfare regimes
Source: Eur J Public Health. 2014 Feb 25;24(3):364–70. doi: 10.1093/eurpub/cku017 (PMC4032483; doi:10.1093/eurpub/cku017)
Supplement: Supplementary Data [file supp_cku017_ejph-2013-07-om-0556-File006.doc]

**Supplementary Material**

**Supplementary Box 1: CASP-12 survey items**

**CASP-12 survey questionnaire items**

1. How often do you think your age prevents you from doing the things you would like to do?
2. How often do you feel that what happens to you is out of your control?
3. How often do you feel left out of things?
4. How often do you think that you can do the things that you want to do?
5. How often do you think that family responsibilities prevent you from doing what you want to do?
6. How often do you think that shortage of money stops you from doing the things you want to do?
7. How often do you look forward to each day?
8. How often do you feel that your life has meaning?
9. How often, on balance, do you look back on your life with a sense of happiness?
10. How often do you feel full of energy these days?
11. How often do you feel that life is full of opportunities?
12. How often do you feel that the future looks good for you?

Answer: Often, sometimes, rarely or never?

**Supplementary Table S1: Age-adjusted slope indices of inequality for CASP-12 by measure of socioeconomic position and welfare regime**

|  | **Southern** | | **Scandinavian** | | **Post-communist** | | **Bismarckian** | |
| --- | --- | --- | --- | --- | --- | --- | --- | --- |
|  | SII | 95% CI | SII | 95% CI | SII | 95% CI | SII | 95% CI |
| **Men** | | | | | | | | |
| Education level | 4.11 | [3.15,5.07] | 0.02 | [-0.89,0.94] | 3.76 | [2.47,5.04] | 2.14 | [1.43,2.84] |
| Occupational Position | 3.04 | [2.14,3.94] | 1.51 | [0.63,2.40] | 3.08 | [1.72,4.45] | 1.83 | [1.13,2.53] |
| Current Wealth | 4.20 | [3.42,4.98] | 2.93 | [2.11,3.74] | 3.92 | [2.79,5.06] | 4.07 | [3.44,4.70] |
| Current Income | 3.27 | [2.48,4.05] | 2.53 | [1.70,3.36] | 4.04 | [2.90,5.17] | 2.91 | [2.28,3.55] |
| *N* | 2219 | | 1236 | | 1152 | | 3028 | |
| **Women** | | | | | | | | |
| Education level | 5.51 | [4.46,6.57] | 1.22 | [0.34,2.10] | 4.40 | [3.22,5.58] | 1.67 | [0.99,2.35] |
| Occupational Position | 4.40 | [3.47,5.34] | 2.13 | [1.24,3.02] | 3.30 | [2.08,4.52] | 1.51 | [0.81,2.21] |
| Current Wealth | 4.75 | [3.94,5.56] | 3.20 | [2.41,4.00] | 3.56 | [2.54,4.59] | 3.96 | [3.35,4.56] |
| Current Income | 3.87 | [3.04,4.69] | 2.56 | [1.74,3.37] | 3.51 | [2.48,4.54] | 3.56 | [2.95,4.17] |
| *N* | 2205 | | 1386 | | 1512 | | 3336 | |

CI = 95% confidence intervals; N = Number of individuals; SEP = socioeconomic position; SII = slope index of inequality

**References**
